# Blue inhalers: blowing hot and cold

**DOI:** 10.1038/s41533-016-0008-4

**Published:** 2017-01-30

**Authors:** Salina Ahmed, Hani Salim, Liz Steed, Hilary Pinnock

**Affiliations:** 1 Centre for Primary Care and Public Health, Asthma UK Centre for Applied Research, Queen Mary University of London, London, UK; 2 Department of Family Medicine, University Putra Malaysia, Selangor, Malaysia; 3 Asthma UK Centre for Applied Research, Usher Institute of Population Health and Informatics, The University of Edinburgh, Edinburgh, UK

We read with interest the study reported by Fletcher et al.^1^ published recently in this journal. Whilst we understand the benefits of uniformity in colour and acknowledge that in the UK ‘blue inhalers’ have become synonymous with reliever medication, we would like to highlight alternative meanings that colour may have amongst various ethnic groups, e.g. South/Southeast Asians, Puerto Ricans etc.^2–4^

These ethnic communities tend to explain illnesses and their treatments based on hot and cold health beliefs,^2–4^ which refer to the representative and symbolic power found in hot and cold constructs, rather than physical temperature itself, e.g. in food, weather, colour, medicine, and emotions. Most respiratory diseases, e.g. asthma, are perceived to be cold illnesses triggered by exposure to cold elements, causing an imbalance of hot and cold energies, thus requiring hot treatment in order to restore the original balance.^2,3^

Blue represents cold, raising questions of whether blue ‘cold’ inhalers may be perceived as less effective medications in the context of these beliefs. This is important because medication beliefs are shown to be powerful predictors of adherence behaviour.^2,3,5^ Additionally, this increases the likelihood of some patients using alternative medical treatments (e.g. rubbing hot chest massage ointment for night symptoms).^2,3^ It has been speculated that colours representing heat, e.g. orange inhalers, may be more acceptable in these populations.^2^ Moreover, these beliefs may be strengthened by the feeling of coldness, when inhaled medication touches the back of the throat, which may influence preferences for oral or dry powder devices instead of aerosol medication. One suggested strategy to overcome this is to complement inhaler use with exposure to hot constructs, e.g. tea.^4^

These communal perceptions are further reinforced by their deep historical presence, e.g. Asian health experts who learn these beliefs in childhood and endorse them. Exploration of the implications of hot and cold beliefs in respiratory medicines could provide useful insight into barriers to adherence/acceptance of inhalers amongst ethnic groups and recognising cross-cultural mindsets can contribute to improving patient care and asthma outcomes in multi-ethnic societies.^2–4^
